# Modulating the import of medium-chain alkanes in *E. coli* through tuned expression of FadL

**DOI:** 10.1186/s13036-016-0026-3

**Published:** 2016-04-05

**Authors:** Toby P. Call, M. Kalim Akhtar, Frank Baganz, Chris Grant

**Affiliations:** Department of Biochemical Engineering, Advanced Centre for Biochemical Engineering, University College London, Torrington Place, London, WC1E 7JE UK; Present address: Department of Biochemistry, University of Cambridge, 80 Tennis Court Road, Cambridge, CB2 1GA UK; Present address: Institute of Molecular Plant Sciences, School of Biological Sciences, University of Edinburgh, Edinburgh, EH9 3BF UK

**Keywords:** Alkanes, Transport proteins, Biosensors, Bio-oxidation, Import, Solvent tolerance

## Abstract

**Background:**

In recent years, there have been intensive efforts to develop synthetic microbial platforms for the production, biosensing and bio-remediation of fossil fuel constituents such as alkanes. Building predictable engineered systems for these applications will require the ability to tightly control and modulate the rate of import of alkanes into the host cell. The native components responsible for the import of alkanes within these systems have yet to be elucidated. To shed further insights on this, we used the AlkBGT alkane monooxygenase complex from *Pseudomonas putida* GPo1 as a reporter system for assessing alkane import in *Escherichia coli*. Two native *E. coli* transporters, FadL and OmpW, were evaluated for octane import given their proven functionality in the uptake of fatty acids along with their structural similarity to the *P. putida* GPo1 alkane importer, AlkL.

**Results:**

Octane import was removed with deletion of *fadL*, but was restored by complementation with a *fadL*-encoding plasmid. Furthermore, tuned overexpression of FadL increased the rate of alkane import by up to 4.5- fold. A FadL deletion strain displayed a small but significant degree of tolerance toward hexane and octane relative to the wild type, while the responsiveness of the well-known alkane biosensor, AlkS, toward octane and decane was strongly reduced by 2.7- and 2.9-fold, respectively.

**Conclusions:**

We unequivocally show for the first time that FadL serves as the major route for medium-chain alkane import in *E. coli*. The experimental approaches used within this study, which include an enzyme-based reporter system and a fluorescent alkane biosensor for quantification and real-time monitoring of alkane import, could be employed as part of an engineering toolkit for optimizing biological systems that depend on the uptake of alkanes. Thus, the findings will be particularly useful for biological applications such as bioremediation and biomanufacturing.

**Electronic supplementary material:**

The online version of this article (doi:10.1186/s13036-016-0026-3) contains supplementary material, which is available to authorized users.

## Background

Fossil fuels including oil, coal and gas still serve as our predominant source of energy, meeting over 85 % of our requirements [1]. Environmental, social and ecological issues have spurred on alternative approaches for fulfilling energy demands. In recent years, intensive efforts have been placed on the development of microbial platforms for the production and waste-management of fossil fuel constituents such as alkanes. Numerous studies have shown that microbes can be engineered for the biosynthesis, bioremediation and biosensing of alkanes [2–5]. An important aspect of developing and optimizing such systems is to understand the transport characteristics of the microbial host. However to date, the passage of alkanes across the membranes still remains a poorly understood area.

AlkL, which forms part of the well-characterized alkane degradation pathway in *Pseudomonas putida* GPo1, was identified as the first known bacterial alkane importer [6]. We showed previously that heterologous expression of *alkL* in *E. coli* could improve dodecane (C12) oxidation by up to 2 orders of magnitude [4]. The uptake process was evaluated by using the *P. putida* alkane monooxygenase (AlkB). This inner membrane complex receives electrons from NADH, via rubredoxin reductase (AlkT) and rubredoxin (AlkG), and transfers one oxygen to the alkane substrate while the other is reduced to H_2_O [7]. Ultimately, it oxidizes alkanes to fatty alcohols, fatty aldehydes, and fatty acids and has been investigated industrially for the bioconversion of octane to octanol [8]. In *P. putida*, alkanes oxidized to fatty acids are gradually converted to acyl-CoA by AlkK (FadD in *Escherichia coli*) for entry into the β-oxidation cycle [7, 9].

**Fig. 1:**
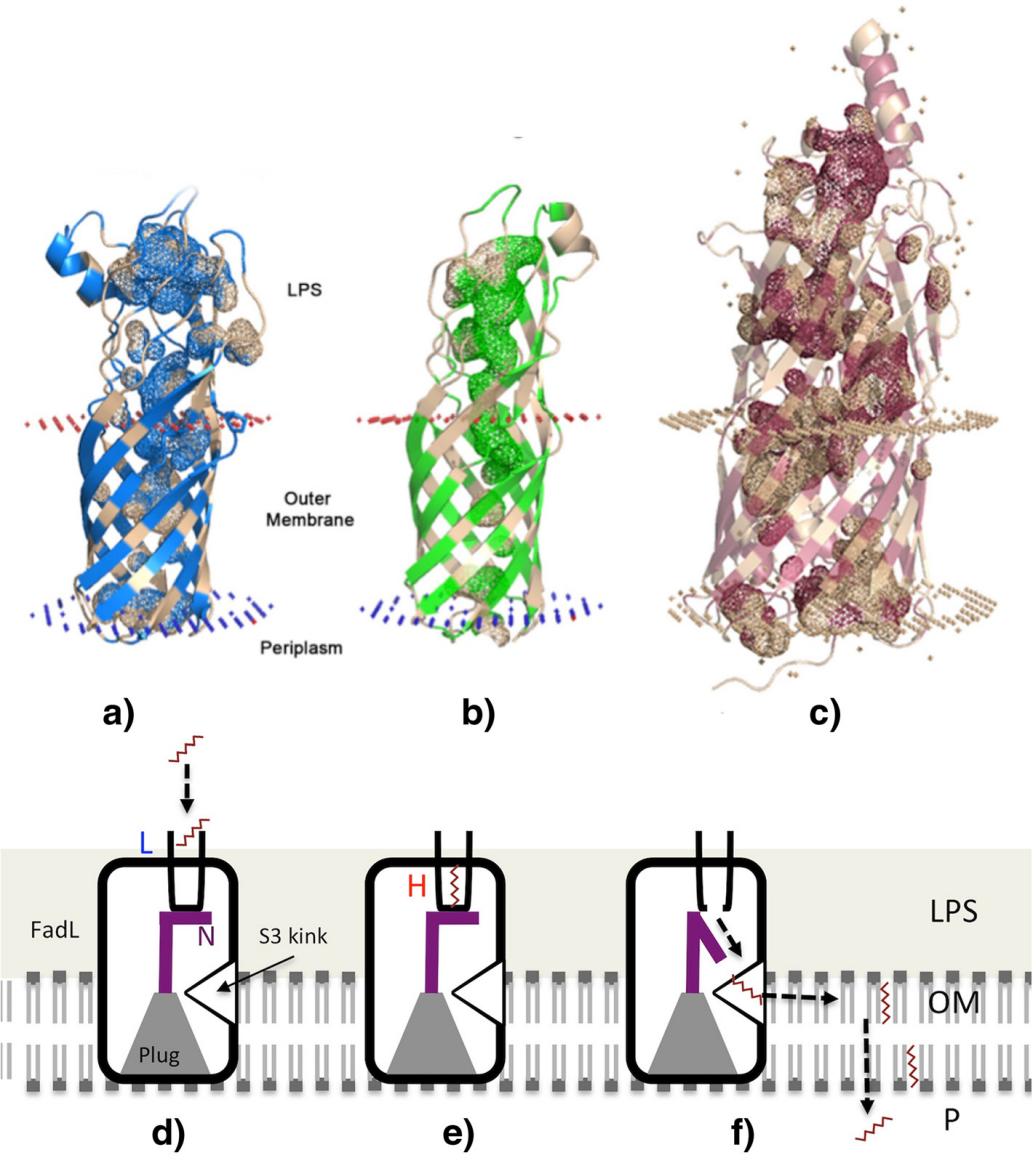
Cartoon structural representations generated in Pymol showing the internal hydrophobic channels. **a** AlkL, **b** OmpW, and **c** FadL spanning the outer membrane. The coloured areas represent hydrophobic regions, including the internal hydrophobic channels leading to the gap in the β-sheet wall. In a model for the mechanism of lateral diffusion of small hydrophobic substrates through FadL: **d** extracellular substrates, in this case octane, bind sequentially to a low affinity binding site (L); **e** then a high affinity binding site (H), **f** leading to conformational change at the N-terminus (*purple*) of the plug. This creates a continuous channel through which the substrate can diffuse through to an opening caused by a kink in the S3 β-sheet strand and into the outer membrane

It is known from previous studies that *E. coli* is capable of medium chain alkane import [10, 11]. We suspected two possible transporters. The first was the OmpW beta barrel outer membrane channel in *E. coli*, which has 27 % sequence homology to AlkL [6]. The second was the outer membrane protein FadL, which is the principal route for aliphatic fatty acid import [12, 13] and shares some structural similarities to AlkL (Fig. 1). All three transporters: AlkL, OmpW, and FadL possess a similar lateral transfer mechanism that allows the entry of small hydrophobic molecules into the outer membrane through an opening in the barrel wall [4, 14, 15] (Fig. 1a-c). In the case of FadL, sequential binding of long-chain fatty acids (LCFAs) with low- and high-affinity binding domains results in a conformational change of FadL (Fig. 1d). The plug domain is eventually displaced (Fig. 1e), forming a passage through a conserved kink in the β-sheet wall [16] that allows lateral diffusion of the LCFA into the outer membrane (Fig. 1f). Within the hydrophobic binding pocket of FadL, the positively charged residues of Arg^157^ and Lys^317^ most likely confer specificity to LCFAs by binding to the negatively charged carboxyl head group [17]. Once in the outer membrane, LCFAs are thought to be adsorbed to the inner membrane and ‘flip-flop’ to its cytosolic face; however the precise route still remains to be elucidated [18].

**Fig. 2:**
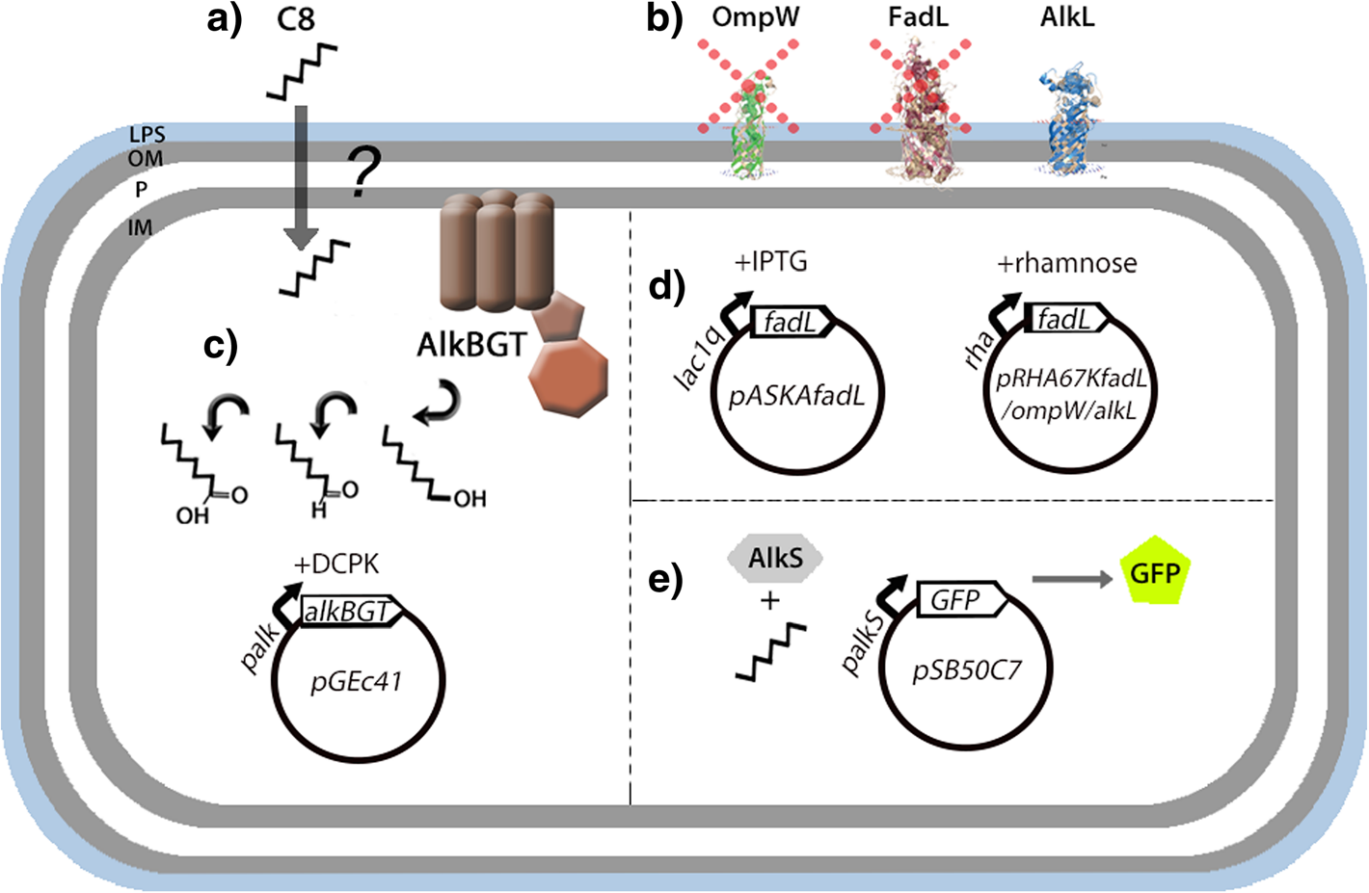
Model system for evaluating (**a**) short chain alkane uptake in *E. Coli*. **b** Knockout strains for candidate transporters OmpW and FadL were selected from the Keio collection. **c** The AlkBGT alkane monooxygenase complex from *P. putida*, encoded on pGEc41, was used as a reporter for alkane import, allowing quantification of intracellular alkane oxidation to alcohols, aldehydes, and fatty acids by gas chromatography. **d** Complementation of *fadL* in the Δ*fadL* knockout strain to restore phenotype was performed with IPTG inducible vector pASKAfadL, whereas the rhamnose inducible vectors: pRHA67KfadL, pRHA67KompW, and pRHA67KalkL permitted fine tuning of overexpression to optimize alkane import via FadL, OmpW, and known alkane importer AlkL. **e** pSB50C7, harboring an alkane inducible AlkS-GFP fluorescence biosensor, was used as a independent reporter system to confirm the results from AlkBGT alkane oxidation in the Δ*fadL* strain

In this study, we investigated OmpW and FadL as potential transporters for the native import of alkanes in *E. coli* using a range of experimental approaches (Fig. 2). We identified the major route of alkane import in *E. coli* and highlight the importance of modulating expression levels of membrane transporters for optimal alkane uptake. Furthermore, we show that FadL can significantly alter the responsivity of alkane biosensing in whole-cell systems in addition to improving alkane tolerance. Overall, this study provides a generalizable framework for the evaluation and characterization of alkane import in whole-cell systems.

## Results and discussion

### Import of octane in *E. coli* is mediated by FadL

To determine whether OmpW and FadL transporters might be involved in the native uptake of octane, the Δ*fadL* and Δ*ompW* strains from the Keio *E. coli* knockout collection [19] were transformed with the plasmid pGEc41 [8] encoding the alkane inducible *alkBGT* alkane monooxygenase complex. With this plasmid, cultivation in the presence of 30 % (v/v) octane leads to the formation of three oxidized products: 1-octanol, 1-octanal and 1-octanoic acid, as reported earlier [4]. The synthesis of 1-octanol and 1-octanal is attributed to AlkB while the synthesis of 1-octanoic acid is catalyzed either by AlkB and/or native enzymes that have yet to be identified.

**Fig. 3:**
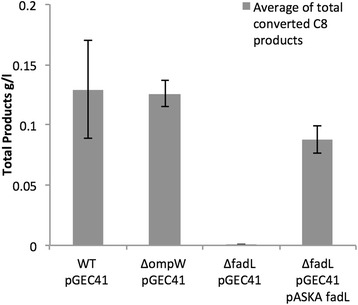
Impact of *E. coli* FadL and OmpW mutants on the whole-cell bioconversion of octane. Experiments were performed in BW25113 single deletion mutants and strains complemented with ASKA plasmid encoding for *fadL* and *ompW*. The total levels of oxidized products was determined by GC-FID after 8 h in partially sealed wells using octane as substrate*.* Results are an average of at least 3 biological replicates and error bars show the standard error

We had shown previously [4] that octane uptake into the cell was the rate limiting step in this bioconversion. By using an excess of octane substrate to overcome mass-transfer rate limitations, we were able to assess the import of octane based on its conversion to the three aforementioned oxidized products. After 8 h of cultivation in a sealed deep-well plate, the total level of oxidized products (1-octanol, 1-octanal and 1-octanoic acid) was separated and quantified by gas chromatography. The Δ*ompW* pGEc41 strain (126 ± 10 mg oxidized products/l) showed no significant reduction compared to the wild type strain (129 ± 40 mg/l of oxidized products), while greatly reduced levels were observed for the Δ*fadL* counterpart (0.34 ± 0.1 mg/l) (Fig. 3). Complementation of Δ*fadL* with a plasmid encoding FadL from the ASKA *E. coli* ORF collection, pASKAfadL, restored conversion of octane to 88 ± 16 mg/l, providing strong initial evidence that octane import in *E. coli* proceeds via FadL.

This has interesting implications in our current understanding of the substrate binding of FadL which has long been known to play a physiological role in the import of long chain fatty acids in *E. coli* [12, 13]. Previously, Black et al. [20] had shown that deletion of the His^110^ residue could significantly reduce the rate of fatty acid binding and import, indicating its importance in the interaction of the carboxylic group with FadL. From structural studies, the zwitterionic head group of the detergent molecule, lauryldimethylamine-oxide (LDAO) is positioned close to Arg^157^ and Lys^317^ suggesting that these residues are also necessary for carboxylic group interaction [14]. Kinetic studies show an almost 10-fold increase in K_m_ for shorter chain fatty acids (C6-C9) compared to LCFAs (C12-C16) [13]. As octane is an aliphatic hydrocarbon without the negatively-charged carboxylic head group, these results do suggest that the binding of substrates to FadL is not necessarily dependent upon the presence of electrostatic charges.

### Optimal import of alkanes requires tuned expression of protein transporters

We noted a complex interplay between IPTG-based membrane transporter overexpression and octane conversion. For example, transforming *E. coli* HB101 strain with pASKAfadL led to a reduction in the specific activity of octane bioconversion from 6.2 to 4.1 μmol min^−1^g^−1^; specific activity further decreased to 2.1 and then to 0.48 μmol min^−1^g^−1^ with induction of recombinant FadL at 50 μM and 500 μM IPTG respectively (Additional file 1: Figure S1a). By switching to the *E. coli* Keio Δ*fadL* strain complemented with a pASKAfadL, a slight increase in activity from 3.1 to 3.25 μmol min^−1^g^−1^ between 0 and 50 μM IPTG induction was initially observed which drastically decreased to 0.71 μmol min^−1^g^−1^ at the higher concentration of 500 μM IPTG (Additional file 1: Figure S1b). Taken together, these observations corroborate our previous findings in which octane conversion was found to be inversely correlated with AlkL induction [4]. Even though the mode of inhibition remains to be proven, these findings strongly support the notion that high expression level of membrane importers may not necessarily offer a sound approach for achieving optimal rates of bioconversion and could, in fact, be detrimental to the physiology of the host organism.

**Fig 4:**
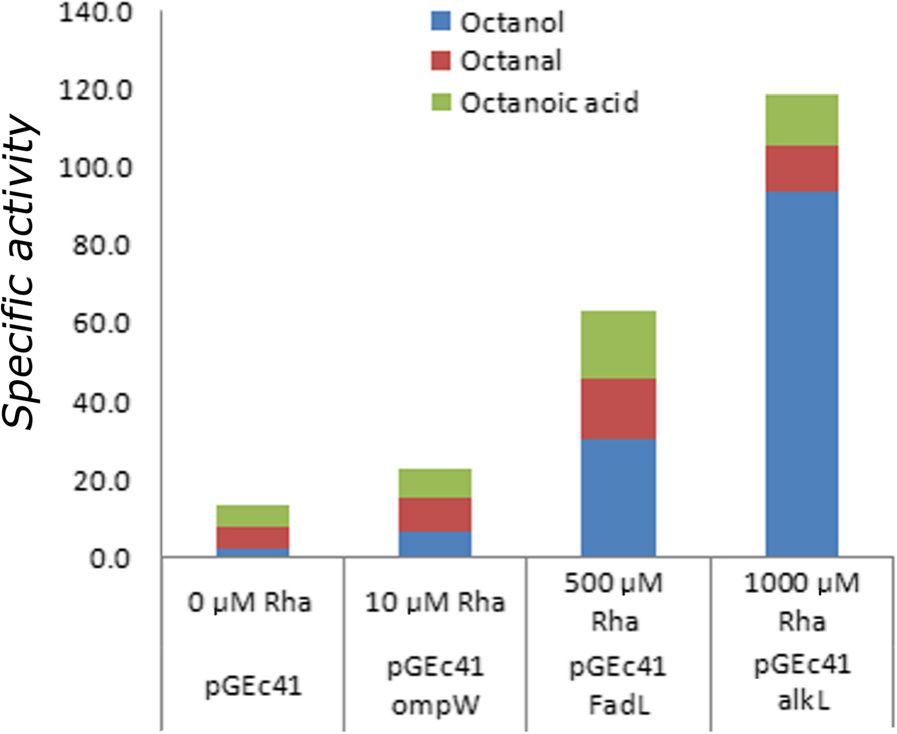
Effect of tightly-regulated expression of *E. coli* FadL and OmpW on the whole-cell bioconversion of octane. Specific maximum specific activity conditions in single well reactions from a multifactorial inducer study (Additional file 1: Figure S2) after 1 h overexpression of OmpW, FadL, and AlkL in the *E. coli* HB101 background strain from an optimized, L-rhamnose inducible vector at optimal inducer concentrations identified for each protein (10, 50, 500, and 1000 μM), compared with wild type strain. AlkB expression was pre-induced with DCPK, and wells were oxygenated and saturated with excess octane substrate (30 % v/v)

In order to precisely evaluate the importance of transporter induction for alkane import, a multifactorial approach was adopted with two important modifications made to the experimental approach. Firstly, vectors pRHA67KompW, pRHA67KalkL*,* and pRHA67KfadL were constructed with the transporters *ompW*, *alkL*, and *fadL*, under control of the *rhaBAD* promoter, previously shown to allow a more tightly controlled expression of the outer membrane protein AlkL [4]. In order to evaluate the performance of the transporters in an industrially relevant context, we tested the genes in the HB101 strain in which we had previously witnessed the highest activity of the alkB enzyme complex. These plasmids were transformed into the wild type HB101 strain, along with pGEc41, and the resulting strains induced with rhamnose concentrations ranging from 0 to 1000 μM. Secondly, the assay was restricted to a one hour growth period within the exponential phase in order to reduce the likelihood of further converting 1-octanol to unknown metabolites by the host organism. ANOVA was applied to the full data set to determine any statistical significance between the variables and the observed outcomes. Highly significant effects on alkane conversion were found for strains harbouring the recombinant FadL and AlkL transporters though not for the OmpW transporter (T ratio of 4.55 and *p* = 0.0002 for FadL) (Additional file 1: Figure S2A), as well as a statistically significant cooperative effect between inducer concentration and transporter induction (T ratio of 3.46 and *p* = 0.0023 for FadL × Inducer concentration Additional file 1: Figure S2B). Specific activity of FadL peaked at 63.1 μmol min^−1^g^−1^ at 500 μM L-rhamnose; this was found to be 2.8 -fold higher than OmpW (22.7 μmol min^−1^g^−1^) at 10 μM L-rhamnose, and 4.5 –fold higher than the wild type at 13.8 μmol min^−1^g^−1^, also treated with L-rhamnose (Fig. 4). Inducing *ompW* expression caused a slight increase at 10 μM followed by a reduction in octane bioconversion at higher L-rhamnose concentrations, similar to the effect seen with FadL and AlkL in the high expression-range construct. These results support the knockout assay demonstrating that FadL, rather than OmpW, serves as the major route for octane uptake in *E. coli* (Fig. 3). The greatest activity, not surprisingly, was observed with the natural alkane importer, AlkL, at 1000 μM L-rhamnose, resulting in a maximum conversion rate of 119 μmol min^−1^g^−1^.

Although the tighter rhaBAD promoter may have helped to resolve leaky or poorly controlled expression issues, intrinsic differences in induction, folding and translocation efficiencies may still have impacted optimal inducer concentration. Furthermore, subtle differences in the affinity of AlkL OmpW, and FadL for octane could also account for the variations in optimal inducer concentrations. Nonetheless, it is clear that optimizing import to maximize conversion rates of octane by AlkBGT not only requires choosing the right expression system, but also sensitive tuning of induction levels.

### Removal of *fadL* confers tolerance toward medium chain alkanes

**Fig. 5:**
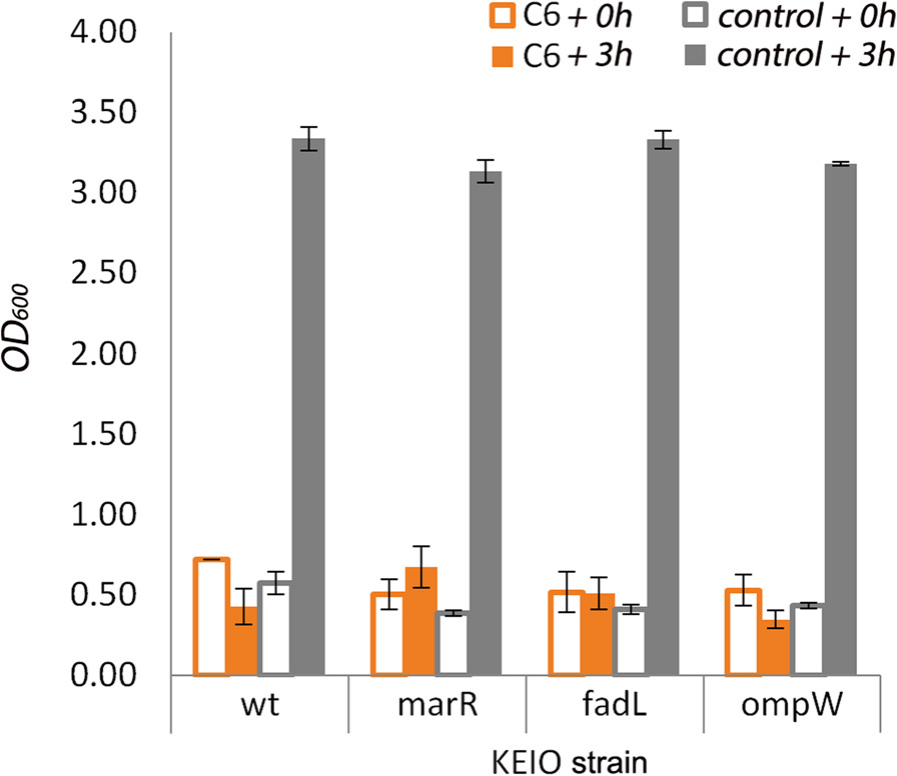
Significance of FadL in conferring tolerance towards alkanes. OD_600_ measurement of initial (outlined columns) cell density of wild-type *E. coli* strain BW25113, Δ*marR,* Δ*fadL,* and Δ*ompW E. coli* strains incubated for 3 h (solid columns) with hexane (10 % v/v, *orange*) compared to controls with no hexane (*grey*). Results are an average of three biological replicates with error bars representing standard deviation

Previously, medium chain alkanes have been reported to exert toxic growth effects [21–23]. In particular, solvents with octanol-water phase partition coefficients (log *P*_*ow*_) between 1.5 and 6.0 are known to alter membrane structure and interfere with respiratory ATP metabolism [24]. Having identified FadL as a significant mediator of octane import, we hypothesized that removal of the import route, through deletion of FadL, may improve tolerance toward medium-chain alkanes such as hexane and octane which have log *P*_*ow*_ values of 3.8 and 4.8, respectively. The cell growths of the wild type, Δ*fadL* and Δ*ompW* strains were therefore assessed by monitoring OD_600_ in the presence and absence of hexane and octane. The Δ*marR* strain was included as a positive control since previous studies had shown it to confer solvent resistance to alkanes including hexane [21, 25, 26]. Growth rates in the absence of solvents were found to be similar, no significant differences in OD_600_ values were noted between all the strains (Additional file 1: Figure S3). However, in the presence of 10 % (v/v) hexane, a clear decrease in cell density was observed for the control and Δ*ompW* strains suggesting loss of cell viability, whereas Δ*fadL* strain neither increased nor decreased in cell density indicating that viability in this particular strain could be maintained (Fig. 5). In contrast and in accordance with previous studies, the Δ*marR* strain was capable of further growth. A similar observation was also made under conditions using 10 % (v/v) octane though the effect was less pronounced, presumably due to the lower cellular toxicity of octane in comparison to hexane (Additional file 1: Figure S4). ANOVA was applied to the dataset to test for statistical significance (Additional file 1: Figure S3). Based on differences in OD values and specific growth rates, a significant interaction between the presence of alkane and the FadL gene knockout (*F* values of 16.26 and 10.42; *p*-values of 0.0004 and 0.002) was observed while differences between replicates were found to be insignificant (*p*-value of 0.58 and 0.78).

Given that the viability of *E. coli* in the presence of high alkane concentration is less affected in a Δ*fadL* genetic background suggests that tolerance could be engineered by reducing or abolishing FadL activity. Such an approach would reduce toxicity by effectively lowering the rate of intracellular influx of alkanes. For the Δ*marR* strain, which was found to possess greater growth resilience in the presence of alkanes, the physiological explanation is probably somewhat more complex since multiple stress-related mechanisms are most likely to operate simultaneously [22]. The induction of the multi-drug efflux pump, AcrAB-TolC, is one such response which is known to confer resistance to multiple drugs, in addition to alkanes including octane [23]. It remains possible that, during the course of this study, the native house-keeping activity of the AcrAB-TolC complex may well have partially masked the tolerance benefit imparted by the deletion of FadL. In light of this, AcrAB-TolC mutants may provide a more ideal genetic background for evaluating the significance of FadL in conferring tolerance toward a range of chemicals including alkanes.

### Impact of native alkane import on whole-cell alkane biosensing

**Fig. 6:**
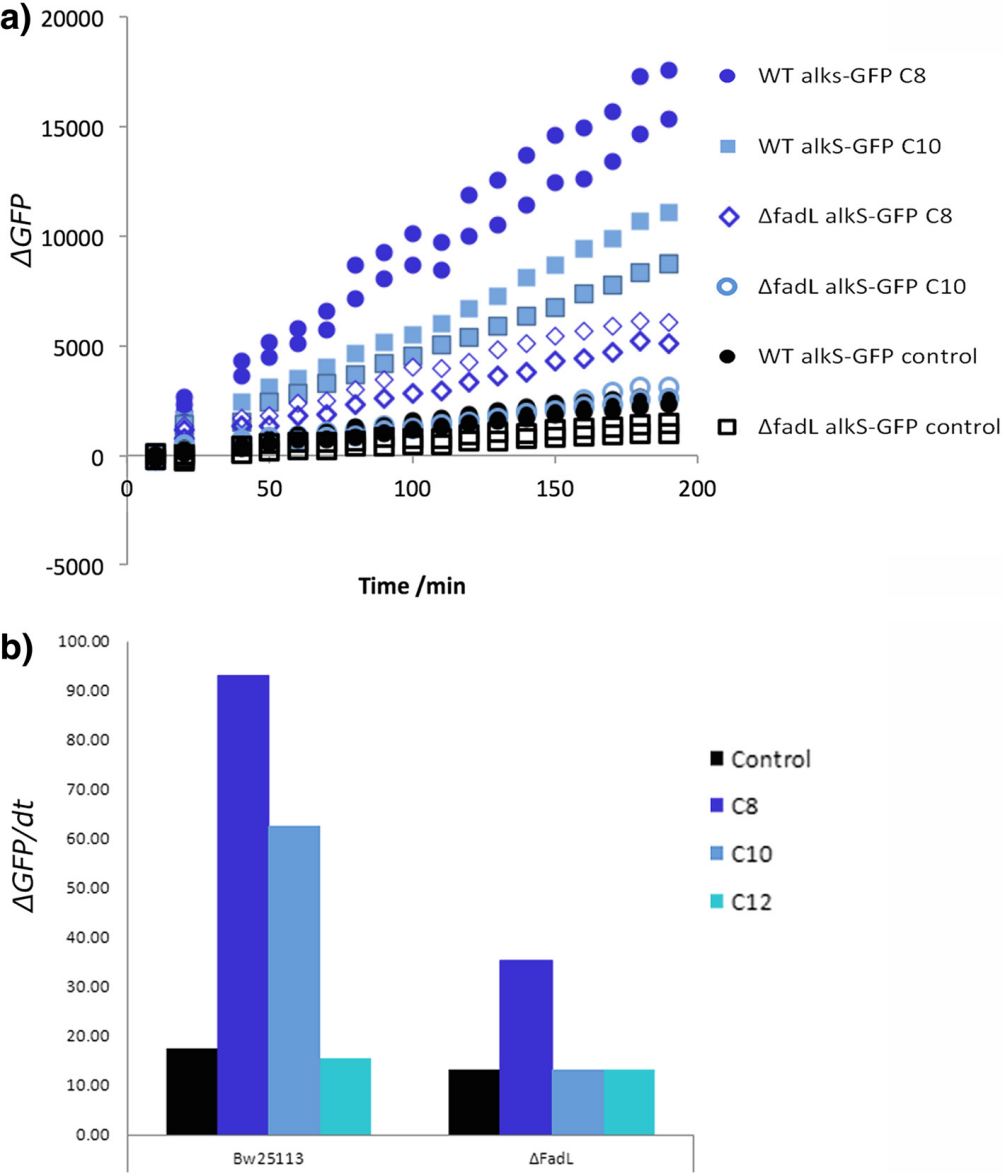
Significance of FadL in for the whole-cell biosensing of medium chain alkanes. **a** Change of fluorescence over 3 h from the wild type BW25113 strain and the Δ*fadL* strain transformed with the alkane biosensor on pSB50C7 and induced using octane, decane, and dodecane. **b** Rate of increase in fluorescence in arbitrary units per minute due to alkane biosensor activation with each alkane, columns display the average of each pair of duplicate conditions at 3 h

In a previous study, we had assembled a whole-cell biosensing system for alkanes by coupling the alkane-binding transcriptional regulator, AlkS from the *P. putida* alkane degradation pathway [24, 27], to a GFP reporter protein and had shown that the signal response for intracellular alkane biosensing could be markedly improved by increasing the expression levels of the AlkL importer. On this basis, we reasoned that if FadL facilitated the import of alkanes then a drop in signal intensity would be expected in a Δ*fadL* background. To experimentally verify this, the AlkS/GFP-based sensor built previously [4] was transferred to both parental *E. coli* BW25113 and Δ*fadL* strains harbouring the pSB50C7 plasmid, and fluorescence was monitored in the presence of octane, decane, and dodecane. As predicted, the fluorescence signal (expressed in arbitrary units) of the Δ*fadL* strain, compared to the parental strain, decreased by 2.7- and 2.9- fold in the presence of octane and decane respectively, thus corroborating the hypothesis that FadL can mediate the import of medium chain alkanes (Fig. 6a and b). By contrast and as expected, there was no significant fluorescence decrease in the presence of dodecane since AlkS acts specifically on C6-C11 alkanes [24, 27]. Further work to engineer an intracellular alkane biosensor with broader specificity could be used to investigate and extend the range of chain length specificity of FadL alkane import.

Controlling the import of signal-eliciting molecules will be important for the development of advanced genetic circuits (including biosensing systems) that respond to external stimuli in a precise, predictable and dose-dependent manner [28]. In this regard, transmembrane importers such as FadL could serve as critical targets for engineering purposes given their importance in influencing the signal response of whole-cell biosensing systems.

## Conclusion

In conclusion we show FadL to be the principle route for octane import in *E. coli*. This study provides a useful toolkit of experimental approaches for evaluating alkane import, and highlights the importance of native outer membrane importers such as FadL for the purpose of developing and optimizing biological applications such as bioremediation and biofuels.

## Methods

### Bioinformatics

Initial identification of candidate genes was expanded from the literature search using online resources: Ecocyc database, (http://www.ncbi.nlm.nih.gov/pmc/articles/PMC99147/). OmpW was previously identified as a target via BLAST searching, revealing identity with the known alkane importer AlkL [4]. Structural alignment and viewing of FadL, AlkL and OmpW was performed using Pymol. The AlkL models were generated using Swiss Model [29, 30], using the 2f1t x-ray crystal structure as a template. Membrane topology was predicted using the orientations of proteins in membranes database [31].

### Strains

The Keio collection, a knockout library of all non-essential genes in *E. coli* [19], and ASKA collection, a clonal plasmid library of all individual *E. coli* gene open reading frames [32] were obtained from the NBRP, Nara University of Science and Technology. Both collections are derived from the *E. coli* K-12 strain and allow systematic studies of gene function based on gene overexpression or complementation. The Keio strains have each gene replaced on the chromosome by a kanamycin resistance gene flanked by FRT recombination sites for removal if necessary. The ASKA strains harbor plasmids with the open reading frame (ORF) for each gene, expressed under control of the LacIa promoter, and a chloramphenicol resistance marker. See Additional file 1: Table S1 for a list of all strains used in this study.

### Plasmid construction

The pGEc41 plasmid is 51.6 kb in size and is constructed from a 21 kb vector pLAFR1 containing a 30 kb fragment with *alk* operon genes *alkBFGHJKL* and *alkST* from *Pseudomonas putida* with the omission of *alkJ* (an alcohol dehydrogenase), *alkK* (an acyl-CoA synthetase) and *alkL* [33]*.* Gene induction was placed under the control of the AlkS – pAlkB promoter system. ASKA plasmids containing the ORF for each target gene were isolated from the ASKA collection [32]. They contain ORFs for all *E. coli* genes cloned between *Sfil* restriction sites, and under the control of isopropyl-β-D-thiogalactopyranoside (IPTG) inducible *lac1q* promoter. See Additional file 1: Table S1 for a list of all plasmids used in this study.

### Media preparation

Lysogeny Broth (LB) growth medium; composed of yeast extract 10 g/L, tryptone 20 g/L and NaCl 20 g/L; was used for most overnight inoculations, Octane uptake and conversion assays were performed in ‘Wubbolts’ enhanced conversion media, taken from Wubbolts et al. [34]. The composition of Wubbolts medium is as follows: KH_2_PO_4_, 4 g/L; K_2_HPO_4_ (3H_2_O), 15.9 g/L; Na_2_HPO_4_ (12H_2_O), 7 g/L; (NH_4_)_2_SO_4,_ 1.2 g/L; NH_4_Cl, 0.2 g/L (all from Sigma Aldrich); yeast extract, 5 g/L*;* L-leucine, 0.6 g/L; L-proline, 0.6 g/L; thiamine, 5 mg/L. The following components were autoclaved and added separatelyo add post autoclaving: MgSO_4_ (7H_2_O) (BDH), 1 g/L (BDH); 1 ml of trace minerals (composition below); 1 ml of 4 % (w/v) CaCl_2_ (2H_2_O) (Alfa Aesar) and 10 g/L D-glucose, were added having all been heat sterilized separately. Filter- sterilized antibiotics were added as appropriate. The trace minerals solution contained per litre of 5 M HClL: 40 g FeSO_4_ (7H_2_O), 10 g MnSO_4_ (H_2_O), 4.75 g CoCl_2_ (6H_2_O), 2 g ZnSO_4_ (7H_2_O), 2 g MoO_4_Na_2_ (2H_2_O), 1 g CuCl_2_, (2H_2_O) and, 0.5 g H_3_BO_3_. Antibiotics were used at the following working concentrations: tetracycline (10 mg/L), chloramphenicol (25 mg/L), Zeocin (100 mg/L) and kanamycin (30 mg/L).

### In-vivo conversion of octane

Comparative bioconversion assays were carried out in 24 deep-well Teflon plates to reduce the adsorption of product and substrate on the on the walls of individual wells. Strains being tested were inoculated overnight in 5 ml LB in 50 ml Falcon tubes with relevant antibiotics for all strains and plasmids. At least 3 biological repeats were isolated from the original transformation plate for each plasmid and condition tested. 1 ml culture volumes were transferred to each well accordingly, and 300 μl of octane (Sigma-Aldrich) was added at *t* = 0. The initial assay (Fig. 3) was carried out in LB media in plates sealed with aluminium foil and clamped with a film cover (EnzyScreen) to counter octane evaporation, and without pre-induction of pGEc41. For overexpression of *ompW* and *fadL*, appropriate concentrations of IPTG were added prior to overnight culture and octane addition. For later assays (Additional file 1: Figure S2 and Fig. 4), 300 μl of substrate was deemed sufficient to counter the effects of evaporation and ensure that substrate availability was not rate-limiting. Gas transfer was facilitated, via a pinhole in the sandwich cover. For pre-induction of pGEc41 in Additional file 1: Figure S2 and Fig. 4, strains were sub-cultured 1:20 in 1 ml of enhanced conversion media (Wubbolt’s media) for 1 h and then induced with 0.05 % (w/v) Dicyclopropylketone (DCPK) and incubated at 37 °C for a further 4 h. For expression from the pRHA67k-derived plasmids, L-rhamnose was added after 1 h of growth in Wubbolt’s media and 4 h prior to octane addition. Assay plates were clamped shut in the shaker incubator (Kuhner) at 37 °C and shaken at 250 RPM with a throw diameter of 25 mm. Well contents were harvested after 8 h (Fig. 3 and Additional file 1: Figure S1) and 1 h (Fig. 4 and Additional file 1: Figure S2) and transferred to pre-weighed 2 ml Eppendorf tubes, centrifuged at 13,000 RPM for 5 min and the supernatant poured into separate tubes. After addition of 800 μl ethyl acetate (Sigma-Aldrich) to separate tubes containing the supernatant and cell pellet, each was vortexed for 1 min to separate the alkanes along with the alkane-derived oxidized products, and then centrifuged for 1 min to separate the aqueous and organic phases. A 600 μl volume of the top phase (organic phase) was transferred to glass vials for analysis by GC-FID.

### Tolerance assays

Seed cultures of the strains were grown overnight in 96-well DSW plates and sub-cultured (using a 5 % (v/v) inoculum) into fresh LB media at 37 °C, shaking at 250 rpm (25 mm amplitude) with absorbance readings monitored at 600 nm using a BMG Clariostar plate reader after 0 h and 2 h. Half the cultures were spiked with an excess of 10 % v/v *n*-alkane and incubated for a further 3 h until the alkane had evaporated, after which point a final set of OD readings was taken.

### Metabolite analysis

Determination of alkanes and their respective oxidized products in microwells was achieved via sacrificial sampling and extraction of both phases into ethyl acetate directly. 800 μl of ethyl acetate was added to the two-phase supernatant following centrifugation at 13,000 rpm for 5 min in a microfuge. The samples were vortexed for 3 × 20 s, prior to centrifugation for 1 min and removal of 100 μl of the organic phase, and then analysed by GC-FID using a Perkin Elmer Autosystem XL equipped with a SGE BPX5 (30 m long; 0.53 mm internal diameter, 1 μm film) capillary column and helium as the carrier gas under a constant pressure of 4 PSI. The proportion of organic phase was determined by cross-referencing the n-alkane peak size to a calibration curve of known standards. Alkanes along with their oxidized derivatives were separated and quantified using the following GC-FID method. For n-octane oxidation product determination the samples were eluted at 70 °C followed by a linear increase of 5 °C minute^−1^ to reach a final temperature of 145 °C. All standards were purchased from Alfa Aesar at the highest purity available (>98 %).

### Determination of dry cell weight

Cell density measurements were taken by sacrificing the well contents and centrifuging the biphasic samples at 13,000 rpm (19,000 g) for 5 min, marking the aqueous volume on the side of the graduated Eppendorf tube; pellets were washed with Tris–HCl pH7.4 and dried in an 80 °C oven until a constant mass was reached.

### Alkane biosensor assay

*E. coli* BW25113 and the Keio Δ*fadL* strain were transformed with an alkS-Superfolder GFP biosensor on pSB50C7. The strains were grown up overnight from single colonies in parallel in deep square well plates (1 ml per well) at 37 °C and 250 rpm (25 mm amplitude) in Luria broth containing 5 g/L glucose and then sub cultured (4 % incoculum) with 10 g/L glycerol added, grown for 2 h, and induced using 3 mM-arabinose. After 3 h growth, 1%v/v alkane substrates were added and grown for 3 h, analysing fluorescence every 10 min (excita- tion 485 nm; emission 517 nm) using a BMG Clariostar plate reader.

## Additional file

Additional file 1: Supplementary Information [35, 36]. (DOCX 25764 kb)
