# Supplementary material for: Modulating the import of medium-chain alkanes in E. coli through tuned expression of FadL
Source: J Biol Eng. 2016 Apr 5;10:5. doi: 10.1186/s13036-016-0026-3 (PMC4822313; doi:10.1186/s13036-016-0026-3)
Supplement: Additional file 1: — Supplementary Information [35, 36]. (DOCX 25764 kb) [file 13036_2016_26_MOESM1_ESM.docx]

**Supplementary information: Modulating the import of medium-chain alkanes in *E. coli* through tuned expression of FadL**


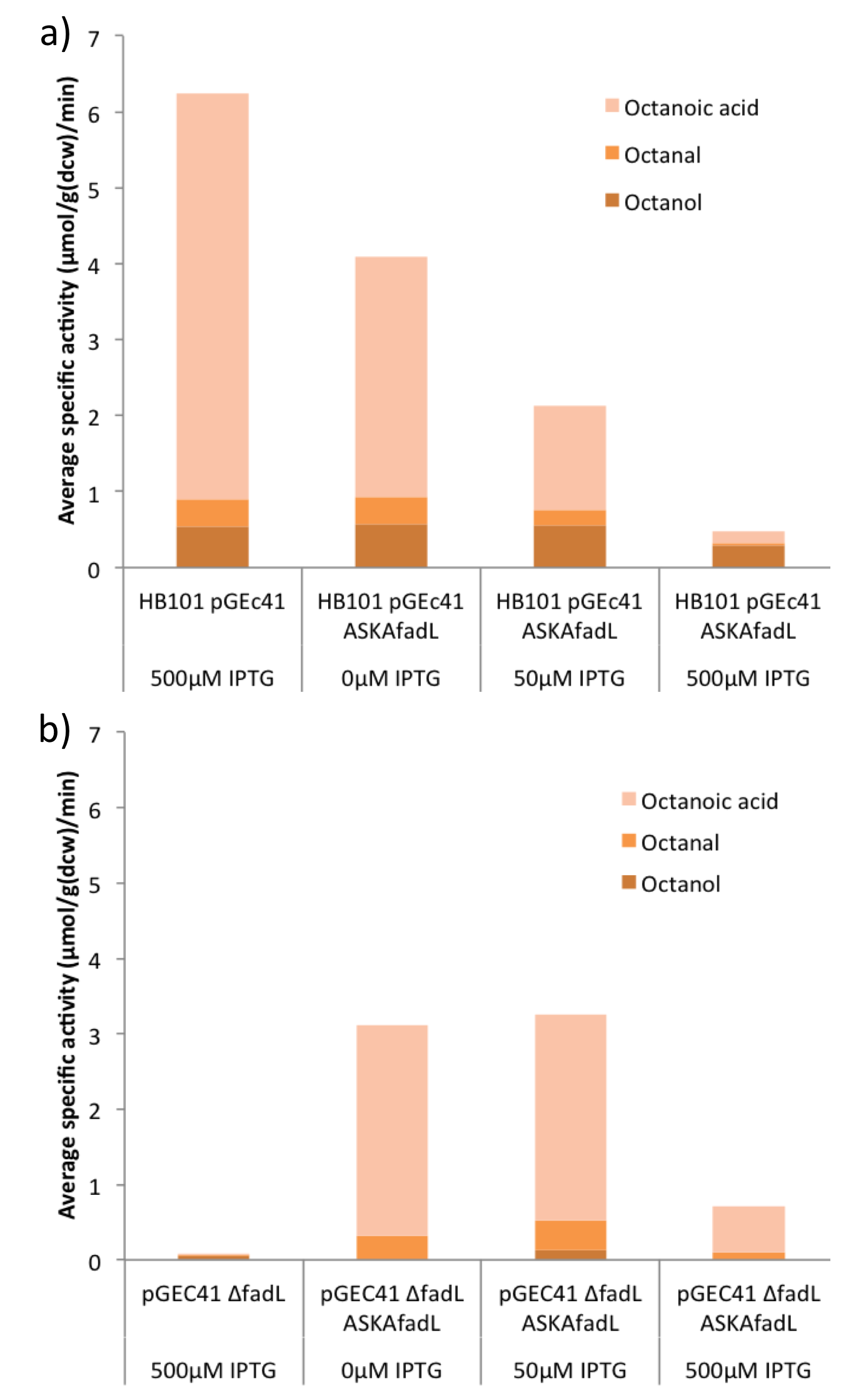


**Fig. S1:** A) Average specific activity over 8 hours for conversion of octane to oxidized products in *E. coli* HB101 control, and the overexpressed ASKA *fadL* plasmid. B) The Keio K12 Δ*fadL* strain rescued with ASKA *fadL* plasmid, and induced with 0, 50, and 500 μM of IPTG. Results are an average of 3 biological replicates from the same bioconversion assay.


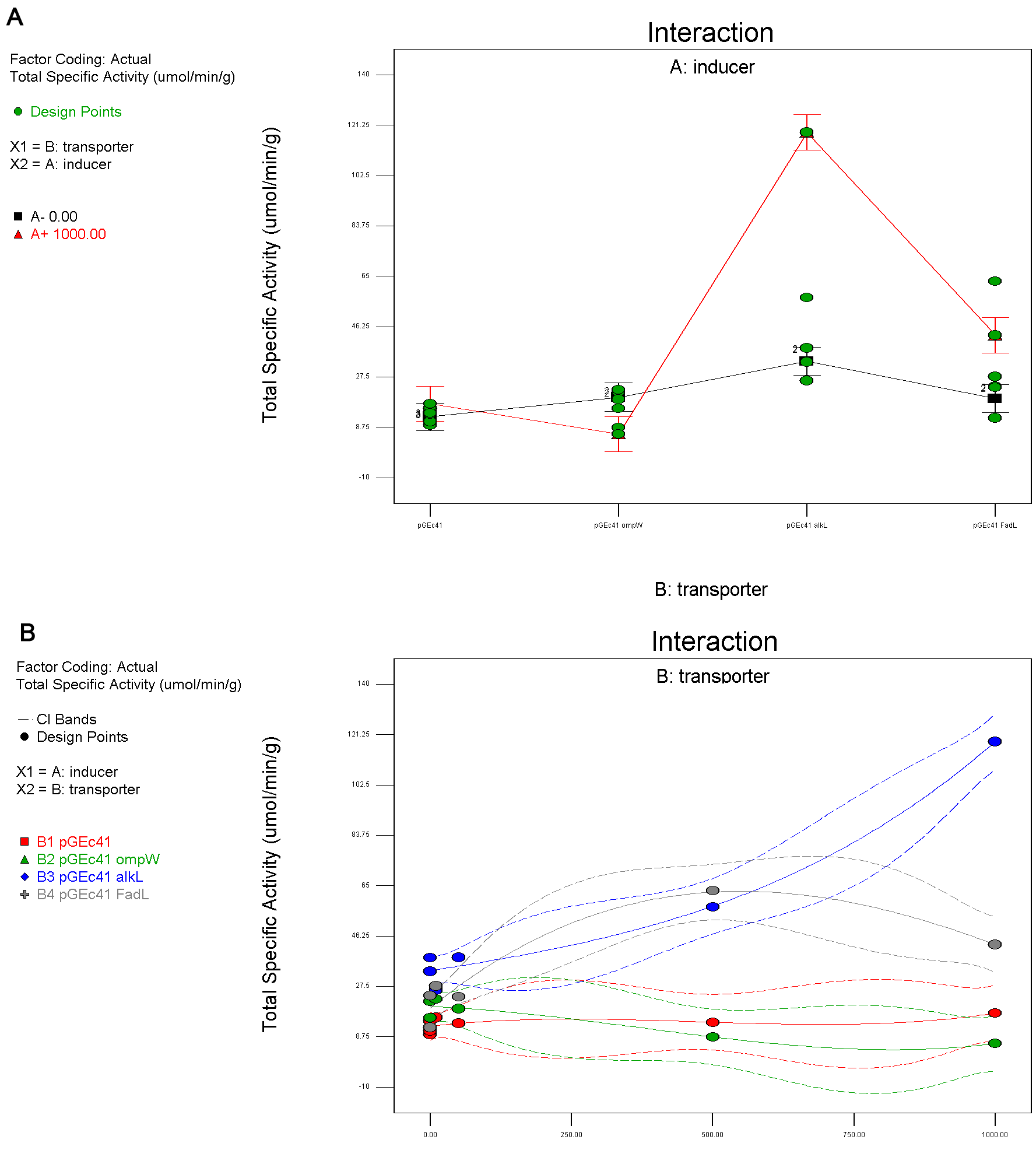


**Fig S2:** Plot of multifactorial interactions affecting specific activity of AlkB octane conversion. A) Between induction levels of the L-rhamnose inducible rhaBAD promoter in pRHA67K *ompW*, pRHA67K *alkL,* and pRHA67K *fadL* at 0, 10, 50, 500, and 1000 μM in the HB101 pGEc41 background. The black dotted line shows the effect of 0 μM induction across each strain, and the red dotted line the effect of 1000 μM. Error bars represent the least significant difference. B) Between strains and three inducer concentrations, 0 μM, 500 μM, and 1000 μM.


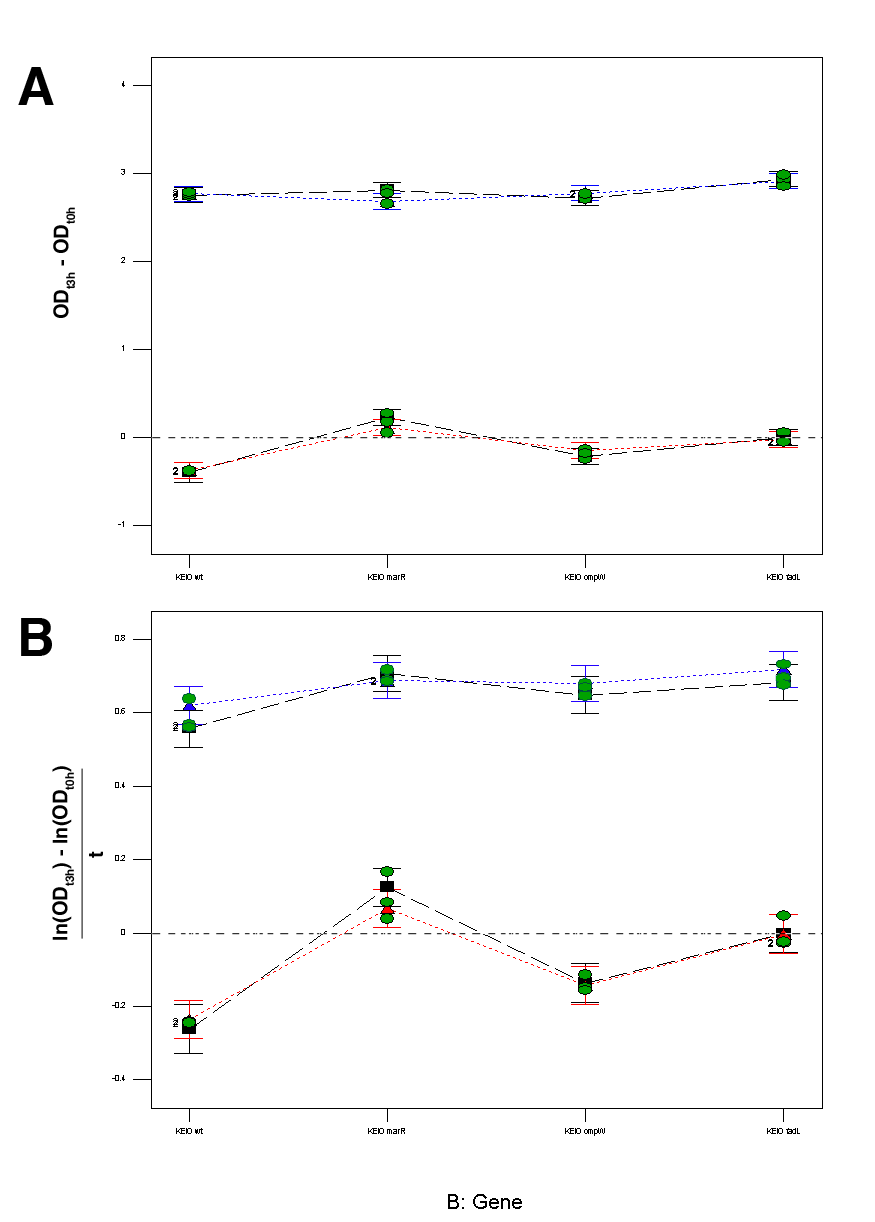

**Fig. S3:** Graph A represents the difference between OD before and after alkane was added (or the equivalent growth time of 3h for the control); graph B shows the specific growth rate over this time. The black and red lines representing the variability between replicates for the cultures grown in the presence of octane and the black and blue lines representing the control cultures grown without any alkane present. Error bars indicate the least significant difference.


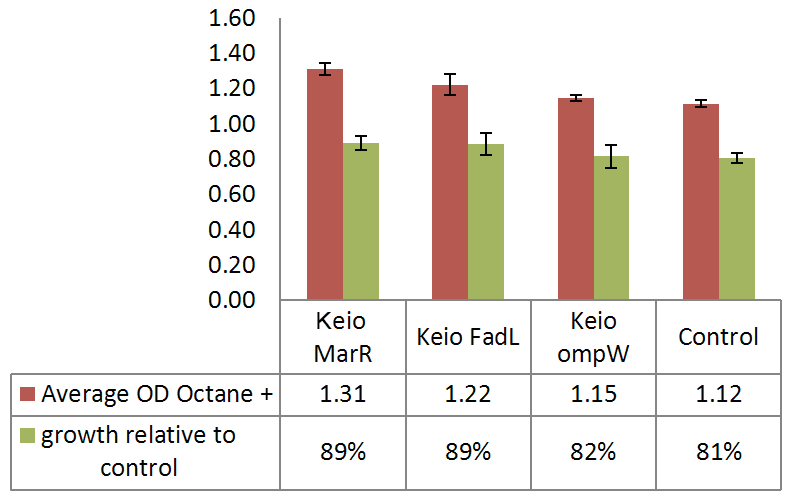


**Fig. S4:** OD_600_ of wild-type, Δ*marR,* Δ*fadL,* Δ*ompW* *E. coli* strains incubated for 3 hours in LB media without octane (red columns) and with 10% v/v octane (green bars). The average OD_600_ value is shown, as well as the growth of octane incubated samples as a percentage of the same strain without octane. Results are an average of 4 biological replicates and error bars represent standard deviation about the mean.

**Table S1:** List of Strains and plasmids used in this study:

| Strains and plasmids | Features | Source/reference |
| --- | --- | --- |
| *Strains:* |  |  |
| *E. coli* HB101 | Hybrid of E. coli K12 and E. coli B | Boyer *et al.* (1969) [35] |
| *E. coli* BW25113 | K12 derivative: parental strain of Keio collection; | Baba *et al.* (2006) [19] |
| *E. coli* BW25113 Δ*fadL*  *E. coli* BW25113 Δ*ompW*  *E. coli* BW25113 Δ*marR* | fadL knockout  ompW knockout  marR knockout | Baba *et al.* (2006) [19]  Baba *et al.* (2006) [19]  Baba *et al.* (2006) [19] |
|  |  |  |
| *Plasmids:* |  |  |
| pGEc41 | Harboring alkane degradation cluster from OCT plasmid: AlkBGT alkane monoxygenase complex | Eggink *et al.* (1987) [10] |
| pSB50C7 | pAlkb - AlkS-sfGFP biosensor | Grant *et al. (*2014) [4] |
| pASKAfadL | fadL gene under control of pLac- *lacI^q^;*with trrnB terminator. Expression vector based on pCA24N | Kitagawa *et al.* (2006) [32] |
| pASKAompW | As above expressing ompW gene | Kitagawa *et al.* (2006) [32] |
| pRHA67KfadL | fadL expressed under control of pRhaBad RhaS-RhaR. Overexpression vector based on pRHA67K with modified RBS and soxR terminator | This study |
| pRHA67KompW | ″ | This study |
| pRHA67KalkL  pRHA67K | ″  pRhaBad RhaS RhaR overexpression vector | Grant *et al. (*2014) [4]  Giacalone *et al.* (2006) [36] |
|  |  |  |

**Supplementary information references:**

4. **Grant, C., Deszcz, D., Wei, Y.-C., Martínez-Torres, R. J., Morris, P., Folliard, T., Sreenivasan, R., Ward, J., Dalby, P., Woodley, J. M., & Baganz, F.** Identification and use of an alkane transporter plug-in for applications in biocatalysis and whole-cell biosensing of alkanes. *Scientific Reports*, 2014; *4*, 5844.

10. **Eggink, G., Lageveen, R. G., Altenburg, B., & Witholt, B.** (1987). Controlled and functional expression of the Pseudomonas oleovorans alkane utilizing system in Pseudomonas putida and Escherichia coli. *Journal of Biological Chemistry*, *262*(36), 17712–17718.

19. **Baba, T., Ara, T., Hasegawa, M., Takai, Y., Okumura, Y., Baba, M., Mori, H.** Construction of *Escherichia coli* K-12 in-frame, single-gene knockout mutants: the Keio collection. *Molecular* *systems biology*, 2006; *2*, 2006.0008.

32. **Kitagawa, M., Ara, T., Arifuzzaman, M., & Ioka-nakamichi, T.** (2006). Complete set of ORF clones of *Escherichia coli* ASKA library ( A Complete Set of *E . coli* K-12 ORF Archive ): Unique Resources for Biological Research, 5005; *299*, 291–299.
